# Manipulative Therapy Plus Ankle Therapeutic Exercises for Adolescent Baseball Players with Chronic Ankle Instability: A Single-Blinded Randomized Controlled Trial

**DOI:** 10.3390/ijerph17144997

**Published:** 2020-07-11

**Authors:** Ho-Jin Shin, Sung-Hyeon Kim, Han Jo Jung, Hwi-young Cho, Suk-Chan Hahm

**Affiliations:** 1 Department of Health Science, Gachon University Graduate School, Incheon 21936, Korea; sports0911@hanmail.net (H.-J.S.); q315201@naver.com (S.-H.K.); 2 Graduate School of Integrative Medicine, CHA University, Seongnam 13488, Korea; tokyo015@hanmail.net; 3 Department of Physical Therapy, College of Health Science, Gachon University, Incheon 21936, Korea

**Keywords:** adolescent baseball player, resistance exercise, chronic ankle instability, high-velocity low-amplitude manipulation, manipulation, pain

## Abstract

Manipulative therapies and exercises are commonly used for the management of chronic ankle instability (CAI), but there is no evidence regarding the efficacy of high-velocity low-amplitude manipulation (HVLA) in addition to ankle therapeutic exercise to improve CAI in adolescent baseball players (ABP). To compare the effects of HVLA plus ankle therapeutic exercise and ankle therapeutic exercise alone on ankle status, pain intensity, pain pressure threshold (PPT), range of motion (ROM) of the ankle joint, and balance ability in ABP with CAI, a single-blinded randomized controlled trial was conducted. A total of 31 ABP with CAI were randomly allocated to the intervention (n = 16) or control (n = 15) groups. The intervention group received HVLA plus resistance exercise twice a week for 4 weeks, while the control group received resistance exercise alone. Ankle status, pain intensity, PPT, ROM, and balance ability were assessed before and after the intervention. The American Orthopedic Foot and Ankle Society scores showed significant group and time interactions (total, *p* = 0.002; pain, *p* < 0.001; alignment, *p* = 0.001). There were significant group and time interactions in pain intensity (resting pain, *p* = 0.008; movement pain, *p* < 0.001). For ROM, there were significant group and time interactions on dorsiflexion (*p* = 0.006) and eversion (*p* = 0.026). The unipedal stance of the balance ability showed significant group and time interactions in path length (*p* = 0.006) and velocity (*p* = 0.006). Adding HVLA to resistance exercises may be synergistically effective in improving the ankle status, pain intensity, ROM, and balance ability in ABP with CAI.

## 1. Introduction

In adolescent sports athletes, the lower extremities are involved in 68.71% of sports injuries, followed by the upper extremities (25,27%), the spine (2.57%), and the head (1.99%); the ankle joint is involved in 24.02% of lower extremity injuries [1]. Notably, ankle sprains account for 37.04% of lower extremity injuries in baseball players [2]. This affects athletic performance in competitive sports, e.g., running, dynamic balance, and functional performance [3]. If an appropriate intervention is not applied for the management of an ankle sprain, it can lead to chronic ankle instability (CAI), which results in pain, decreased range of motion (ROM), and limitation of functional movements [3,4,5,6]. CAI can also affect psychological anxiety and quality of life [7,8]. 

Several studies have evaluated the effects of high-velocity low-amplitude manipulation (HVLA), a manipulative therapy for aligning the joint and increasing ROM, on musculoskeletal diseases [9,10,11,12,13]. HVLA increases ROM and alleviates pain after injury. HVLA is effective in pain reduction and ROM improvement in patients with subacute and chronic ankle sprains [12,14]. However, some studies report contradictory results, in that HVLA has no effect on ROM improvement [15,16]. Thus, it is difficult to generalize the clinical use of HVLA for CAI rehabilitation.

Various interventions, such as massage, exercise, and manual therapy, can be applied for CAI management [17,18,19]. Among them, therapeutic exercise is commonly used for CAI rehabilitation, and various exercise protocols exist, such as balance training, strength exercise, and neuromuscular training [10,20,21,22]. Several studies have reported that therapeutic exercise enhances the dynamic and static balance and function of the ankle joint in CAI patients [23,24,25]. Previous studies related to rehabilitation in musculoskeletal diseases have reported that manual therapy combined with therapeutic exercise is more effective in improving physical function than therapeutic exercise alone [26,27]. Considering that HVLA is effective for joint alignment and ROM improvement [12,13,14,15,16], its application followed by therapeutic exercise may have a more positive effect than therapeutic exercise alone. However, no randomized controlled trial has been conducted to investigate the effects of HVLA plus ankle therapeutic exercise in adolescent baseball players (ABP) to improve ankle instability. 

This study aimed to compare the effects of HVLA plus resistance exercise with those of resistance exercise alone on ankle status, pain intensity, pain pressure threshold (PPT), ROM, and balance ability in ABP with CAI. We hypothesized that adding HVLA to ankle therapeutic exercise would have more positive effects on CAI in ABP compared with ankle therapeutic exercise alone.

## 2. Methods 

### 2.1. Design and Ethics

This study was designed as a single-blinded randomized controlled trial. This trial was approved by the Gachon University Institutional Review Board (1044396-201911-HR-197-01) and registered (WHO International Clinical Trials Registry Platform, KCT0004750) before participant enrollment. The content of the study was explained to all the participants in detail and a written informed consent was obtained prior to their enrollment in the study.

### 2.2. Participants

ABP were recruited from multiple organizations (Sungnam Middle School and Sungnam High School located in Dongjak-gu, Dondo Middle School located in Mapo-gu, Seoul, respectively). The inclusion criteria were as follows [28]: (1) active adolescent baseball players with at least 1 year of sports experience, (2) a previous ankle sprain at least 6 months before the study, and (3) score ≤ 25 in the Cumberland ankle instability tool (CAIT). The exclusion criteria were as follows: (1) history of musculoskeletal surgery in the lower extremity and (2) history of ankle sprain in the last 6 weeks. In addition, participant screening included three physical tests (anterior draw test, talar tilt test, and posterior draw test) to confirm instability. 

The sample size was calculated using the computer software G-power 3.1.9.4. In the present study, the effect size was set to 0.25 (medium effect size) [29] and the alpha level was 0.05. Based on these values, 34 participants (17 per group) were required to achieve 80% power using a 2-sided test. With a 10% dropout rate, a total of 38 subjects were required.

### 2.3. Experimental Procedures and Interventions

Participants were randomly assigned to the intervention or control groups using a permuted block (block size: 4) randomization method [30]. The randomization sequence was generated using a permuted block sequence from a random table and participants were randomly allocated to the intervention (n = 16) or control (n = 15) groups by an independent staff member. Ankle status, pain intensity, PPT, ROM, and balance ability were assessed before and after the intervention at Gachon university by two physical therapists who had received a master’s degree and also had clinical experience of more than 7 years. The outcome assessors were not involved in the intervention and were blinded to the group allocation of the participants by concealing the codes of the participant’s group status. Participants were exposed to baseball activities during the experimental period.

The intervention group received the HVLA intervention and both groups received the ankle therapeutic exercise. The HVLA applied to the intervention group proceeded as follows [31]. Participants were instructed to stretch their arms behind them to support the upper body and to stretch out the lower extremity with CAI. The therapist faced the participant’s leg, wrapping the front of the talus with the five fingers of both hands, touching the sole with the thumbs. After firmly wrapping the talus, the therapist slowly pulled it in the caudal direction. HVLA was applied while maintaining traction in the caudal direction at the maximum range of tension.

The ankle therapeutic exercise consisted of a warm-up, main exercise, and cool-down. The warm-up consisted of stretching and mobility exercises, which lasted for 10 min, and the cool-down proceeded in the same way as the warm-up. The stretching was applied to the dorsiflexor, plantar flexor, invertor, and evertor muscles, and maintained for 10 s at the end-range of the ROM. Two sets were repeated for each muscle. The mobility exercise consisted of two sets of the ankle rotation in a standing position for 20 s. The main exercise consisted of dorsiflexion, plantar flexion, eversion, and inversion strength training using a TheraBand in a sitting position (on the floor with the knee extended) [32]. The looped end of TheraBand was placed on the foot (dorsiflexion), under the sole (plantar flexion), outside the foot (eversion), and inside the foot (inversion). The experimenter fixed the TheraBand and instructed participants to perform only the ankle joint movements without the knee and hip joint movements. Each movement was repeated 15 times in one set and three sets were performed for all the movements [32]. The interval between sets and movements was 30 s and 1 min, respectively. The intervention period was 4 weeks, consisting of two sessions per week for a total of eight sessions, and each session lasted approximately 30 min.

### 2.4. Outcome Measures

#### 2.4.1. Primary Variable

To assess ankle status, American Orthopedic Foot and Ankle Society (AOFAS) scores were used [33]. The AOFAS score is a tool used to assess the pain, function, and alignment of the foot and ankle joints in patients with foot and ankle discomforts. The assessor proceeded after explaining in detail the contents of the AOFAS score to the participants. AOFAS-function (gait abnormality, sagittal motion, hindfoot motion, ankle stability) and AOFAS-alignment were measured by the evaluation of the assessor. The AOFAS score ranged from 0 (severe pain and damage) to 100 (no damage).

#### 2.4.2. Secondary Variables

This study used a visual analogue scale (VAS) to quantify pain intensity [34]. All the participants were instructed to check the subjective pain intensity of the ankle joint that they felt between 0 (no pain) and 100 (the maximum pain they could imagine). The assessment was divided into two conditions: resting pain and movement pain. Resting pain is defined as the intensity of pain while resting and movement pain is defined as the intensity of pain induced by movement.

To assess the pain pressure threshold (PPT), a digital algometer (Somedic AB, Farsta, Sweden) was used [35]. The assessor marked the halfway point of the anterior talofibular ligament with a marker as the measurement point. The participants were instructed to maintain a supine position and the assessor applied a 1 cm^2^ probe with a pressure of 40 kPa/s to the measurement point vertically [36]. The assessor applied pressure until the participants expressed pain responses such as frowning, dodging, and noise. The mean value of three repeated measurements was used as the data.

A digital inclinometer was used to measure active pain-free ROM [37]. ROM was measured for dorsiflexion, plantar flexion, inversion, and eversion of the ankle joint. Dorsiflexion and plantar flexion ROM were measured in the prone position, bending the knee at 90°. The axis was set as the lateral malleolus, stationary arm as the fibular head, and moving arm as the metatarsal fifth head. In the supine position, inversion and eversion ROM were measured based on the movement of the metatarsal head. The axis was set as the natural position according to the alignment of the goniometer arm, stationary arm as the midline of the leg, and moving arm as the plantar aspect of the metatarsal head. For all ROM measurements, detailed explanation about the movement was given to all participants and given the verbal cue to “reach the maximum range without pain.” All ROM evaluations were repeated three times and the mean value was used as the data.

Balance ability was assessed using the AMTI AccuSway (Advanced Mechanical Technology, Inc., Watertown, MA, USA) [38]. Assessments were obtained in the bipedal and unipedal stance conditions. Participants stood straight and barefoot on the AccuSway with arms placed across their chest and were instructed to hold their heads as still as possible. They were instructed to face forward and look at an 8 cm × 6 cm X marked at the same height as their eyes on a wall that was 1.5 m in front of them [39]. For the unipedal stance, they stood on the affected side. Measurements began with the cue sign and data were measured at 200 Hz. Measurement continued for 20 s and the value of a total of 10 s, excluding the first and last 5 s, was used as the data. The mean value from three repeated measurements was used as the data. The interval between measurements and conditions was 1 min and 5 min, respectively. The following variables were measured to evaluate the balance ability of the participants: (1) 95% sway area (SA), (2) path length (PL), (3) average radial velocity (V), (4) anteroposterior standard deviation (APSD), and (5) left-right standard deviation (LRSD).

### 2.5. Statistical Analysis

Data were analyzed using SPSS 25.0 software (SPSS Inc., Chicago, IL, USA). A Shapiro–Wilk test was used for testing normality and then data analysis was performed. All primary and secondary variables assessed in this study met the criterion of normality. An independent t-test or chi-square test was performed to compare the general characteristics between the two groups. A repeated measures ANOVA was used to analyze the changes in variables between the groups over time. A paired t-test was used to analyze the change according to time in each group and an independent t-test was used to analyze the difference in change values between the groups. A *p*-value < 0.05 was considered statistically significant. Finally, the effect size (ES) was calculated by Cohen’s d, and the formula is as follows:

(1) $${\mathrm{Cohen}}^{\prime }s\;d=\frac{\bar{x_{1}}-\bar{x_{2}}}{s_{pooled}}$$

## 3. Results

### 3.1. Participants

**Figure 1:**
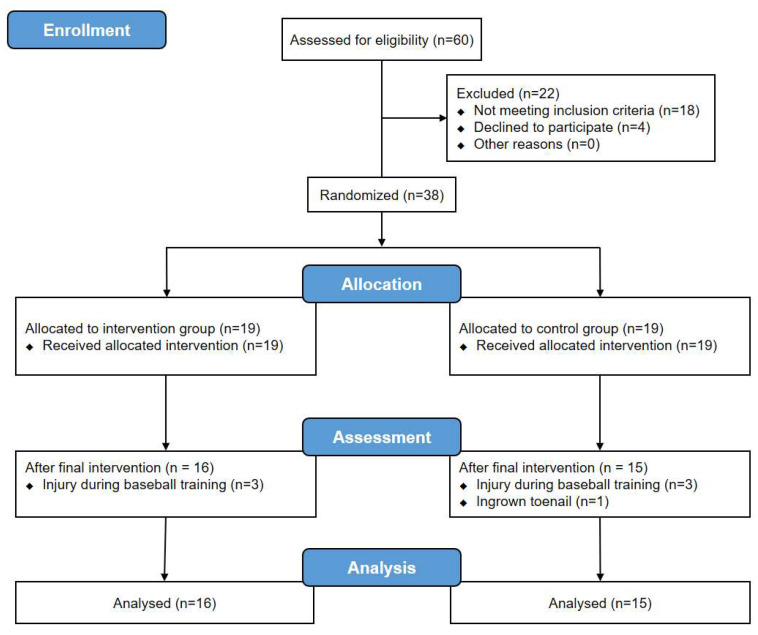
Flow diagram of study participants.

The participants in this study were recruited over 4 weeks from 29 November 2019. The experiment was conducted from 23 December 2019 to 20 January 2020. Sixty male ABP volunteered for the experiment and 36 participants who met the inclusion criteria participated in the study. Five participants dropped out during the experiment and a total of 31 participants fully participated in the experiment. Three participants in the intervention group dropped out due to injury during training, while two participants in the control group dropped out due to injury during training and ingrown toenail surgery, respectively (Figure 1).

There was no significant difference in all the variables (Table 1) including participant age (intervention group: 13.88 ± 1.54, control group: 14.27 ± 1.49), height (intervention group: 166.54 ± 9.60, control group: 169.49 ± 7.82), weight (intervention group: 67.68 ± 13.80, control group: 71.18 ± 16.69), and sports career (intervention group: 3.75 ± 2.27, control group: 4.17 ± 2.31). 

### 3.2. Status of the Ankle

All the variables except AOFAS-function (Table 2) showed significant group and time interactions (AOFAS-total, *p* = 0.002; AOFAS-pain, *p* < 0.001; AOFAS-function, *p* = 0.621; AOFAS-alignment, *p* = 0.001). The intervention group showed significant differences before and after the intervention in AOFAS-total (*p* < 0.001, ES = 1.74), AOFAS-pain (*p* < 0.001, ES = 1.23), AOFAS-function (*p* = 0.001, ES = 1.46), and AOFAS-alignment (*p* = 0.002, ES = 0.97). However, the control group only showed significant differences in AOFAS-total (*p* = 0.031, ES = 0.64) and AOFAS-function (*p* = 0.001, ES = 1.23). Comparison between the groups showed significant differences in AOFAS-total (*p* = 0.001), AOFAS-pain (*p* = 0.001), and AOFAS-alignment (*p* = 0.002).

### 3.3. Pain Intensity and PPT

In the pain results (Table 3), there was a significant group and time interaction in VAS-resting pain (*p* = 0.008) and VAS-movement pain (*p* < 0.001). The intervention group showed significant differences before and after intervention in resting pain intensity (*p* = 0.001, ES = −1.11) and movement pain intensity (*p* < 0.001, ES = −2.89). However, the control group did not show significant differences in pain intensities. Comparison between the groups showed significant differences in VAS-resting pain (*p* = 0.038) and VAS-movement pain (*p* < 0.001). PPT did not show a significant difference in both the intervention and control groups before and after intervention and there was no significant difference between the groups.

### 3.4. Ankle ROM

In the ankle ROM results (Table 4), there was a significant group and time interaction in dorsiflexion (*p* = 0.006) and eversion (*p* = 0.026). The intervention group showed significant differences before and after intervention in dorsiflexion (*p* = 0.006, ES = 0.94) and eversion (*p* = 0.004, ES = 0.56). However, the control group did not show significant differences in any of the variables. Comparison between the groups showed significant differences in dorsiflexion (*p* < 0.001) and eversion (*p* = 0.030).

### 3.5. Balance Ability

In the bipedal stance condition (Table 5), there was no significant group and time interaction for all the variables. Neither the intervention nor the control groups showed significant differences before and after intervention for all the variables. Comparison between the groups did not show significant differences in any of the variables.

In the unipedal stance condition (Table 5), there was a significant group and time interaction in PL (*p* = 0.006) and V (*p* = 0.006). The intervention group showed significant differences before and after intervention in PL (*p* = 0.002, ES = −0.22), V (*p* = 0.002, ES = −0.20), and APSD (*p* = 0.012, ES = 0.21). However, the control group did not show significant differences before and after the intervention in all variables. Comparison between the groups showed significant differences in PL (*p* = 0.007) and V (*p* = 0.007).

## 4. Discussion

The aim of this study was to investigate the effects of 4 weeks of HVLA plus ankle therapeutic exercise and ankle therapeutic exercise alone on ankle status, pain intensity, PPT, ROM, and balance ability of ABP with CAI. The results of this study showed that 4 weeks of HVLA plus ankle therapeutic exercise showed significant improvement in ankle status, pain intensity, ROM, and balance ability in ABP with CAI. Interestingly, the intervention group, which combined HVLA and ankle therapeutic exercise, showed significant improvement in ankle status, pain intensity, ROM, and balance ability compared with the control group. Based on the results of this study, HVLA plus ankle therapeutic exercise is effective for the rehabilitation of ABP with CAI.

In this study, both the intervention and control groups showed improvement in ankle status after the intervention. The intervention group showed an effect size of 1.74 in AOFAS-total, 1.23 in AOFAS-pain, 1.46 in AOFAS-function, and 0.97 in AOFAS-alignment. The control group showed an effect size of 0.64 in AOFAS-total, 0.29 in AOFAS-pain, 1.23 in AOFAS-function, and 0 in AOFAS-alignment. We also found that HVLA plus ankle therapeutic exercise significantly improved ankle pain and alignment compared to the control group. Previous studies reported significant improvements in the physical function of musculoskeletal disorders using manual therapy plus therapeutic exercise [26,27], which may support the results of this study. In addition, although AOFAS score is a typical assessment tool used to assess the quality of the ankle joint status [40,41], no studies have assessed the ankle joint status of ABP with CAI using the AOFAS score. This study is the first randomized controlled trial that verified the effects of HVLA plus ankle therapeutic exercise on the ankle status in ABP with CAI using the AOFAS score. 

The control group in this study did not show significant differences in pain-related variables after the intervention. The results of the control group showed contradictory results from that of the previous study, which applied therapeutic exercise to CAI patients [42]. In the study by Lubbe et al. (2015), therapeutic exercise included peroneal strengthening as well as proprioceptive training and the intervention period was seven sessions per week for 5 weeks, which was relatively much more intensive than in this study. The differences in elements and period of intervention between the two studies would have led to contradictory results. Compared with the control group, the intervention group showed significant improvement in both pain intensity and AOFAS-pain after intervention. The results of previous studies that applied HVLA supported the results of this study, which verified the positive effect of HVLA on pain reduction [42,43]. Such pain reduction may have affected the improvement of the ROM of the ankle joint in this study.

In this study, the intervention group showed a significant improvement in the ROM of dorsiflexion and eversion after the intervention while the control group did not show significant results in all of the ROM after the intervention. CAI shows deficits in ROM, strength, balance, and functional activity [44,45,46]. Owing to these multiple deficits, the rehabilitation configuration for CAI depends on the condition of the participant. For example, the strength deficit requires concentric and eccentric exercise and the ROM deficit requires joint mobilization, manipulation, and stretching to improve ROM [47]. The control group in this study was focused on therapy for muscle strength and joint stability rather than ROM recovery; therefore, no significant improvement was observed in ROM. HVLA may improve the ankle ROM in patients with ankle sprain, which is believed to improve dorsiflexion ROM through posterior gliding of the talus passively [47]. The results of previous studies, which reported the improvement of dorsiflexion ROM by promoting the posterior glide of the talus through manual therapy in CAI patients, support the results of this study [17,31,48,49]. However, some previous studies reported contradictory results where HVLA did not affect the dorsiflexion ROM of the ankle [18,19]. Unlike this study, Nield et al. (1993) conducted a study on asymptomatic participants with normal ankles. Andersen et al. (2003) also conducted a study on participants with a history of ankle sprain, but no current pain and residual symptoms, and obtained measurements immediately after HVLA to confirm the immediate effect of treatment. It seems that the difference in results is due to the period of intervention and characteristics of the participants.

Unlike the study by Hall et al. (2018), which reported the improvement of static balance with therapeutic exercise, the control group in this study did not show an improvement [50]. Hall et al. (2018) applied proprioceptive neuromuscular facilitation and heel raise intervention as well as TheraBand exercise, similar to this study. This study combined ankle muscle strengthening with overall lower extremity muscle strengthening, coordination training by PNF pattern, and heel raise intervention to involve training of postural control in a naturally standing position, which may have led to contradictory results from the control group in this study. Compared to the control group, the intervention group may have had a synergic effect on the proprioceptors located in the ligament and joint capsule of the ankle joint, considering that Holt et al. (2010) reported that HVLA for 4 weeks improved the proprioceptive sense of the ankle joint [51]. HVLA presumably relaxed abnormally shortened muscles to allow normal input of proprioceptive sense and muscular activity.

This study has some limitations. First, although this study showed significant effects of HVLA plus ankle therapeutic exercise on ankle status, pain intensity, ROM, and balance ability in ABP with CAI, the current study assessed the findings only after 8 sessions for 4 weeks. Further studies to investigate the effects of repeated interventions over longer periods are necessary to evaluate the clinical use of the interventions. Second, in this study, follow-up assessment was not performed and retention of the effect of the intervention was not confirmed.

## 5. Conclusions

The results of this study show that the application of HVLA plus ankle therapeutic exercise improves ankle status, pain, ROM, and balance ability in ABP with CAI compared to ankle therapeutic exercise alone. However, further studies in consideration of the study limitations need to be conducted for the clinical application of HVLA combined with ankle therapeutic exercise for the rehabilitation of ABP with CAI.

## Figures and Tables

**Table 1:** General characteristics of the participants.

	Intervention Group (n = 16)	Control Group (n = 15)	*p*-Value
**Age (y)**	13.88 ± 1.54	14.27 ± 1.49	0.478
**Affected Side (%)**			
**Rt**	32.3	29.0	0.886
**Lt**	19.4	19.4	
**Height (cm)**	166.54 ± 9.60	169.49 ± 7.82	0.359
**Weight (kg)**	67.68 ± 13.80	71.18 ± 16.69	0.529
**Body Mass Index (kg/m^2^)**	24.18 ± 3.25	24.50 ± 4.04	0.807
**Career (y)**	3.75 ± 2.27	4.17 ± 2.31	0.616

Values are expressed as mean ± standard deviation or %. Rt, right; Lt, left.

**Table 2:** The changes in AOFAS (American Orthopedic Foot and Ankle Society) scores.

	Pre ^a^	Post ^a^	Within Group ^b^		Between Groups ^b^	*p*-Value (T*G)
			Effect Size	Mean (CI)		
**AOFAS Score-total**						
Intervention Group	65.06 ± 8.42	83.88 ± 12.20	1.74	−18.82 ^†††^ (−25.59, −12.03)	−13.49 ^‡‡^ (−21.52, −5.44)	0.002 **
Control Group	62.60 ± 8.76	67.93 ± 7.80	0.64	−5.33 ^†^ (−10.11, −0.55)		
**AOFAS Score-pain**						
Intervention Group	27.50 ± 4.47	34.38 ± 6.29	1.23	−6.88 ^†††^ (−9.43, −4.32)	−8.21 ^‡‡^ (−11.86, −4.55)	<0.001 ***
Control Group	28.00 ± 4.14	26.67 ± 4.88	0.29	1.33 (−1.53, 4.19)		
**AOFAS Score-function**						
Intervention Group	37.56 ± 6.21	45.50 ± 3.86	1.46	−7.94 ^††^ (−12.00, −3.88)	−1.27 (−6.48, 3.93)	0.621
Control Group	34.60 ± 5.84	41.27 ± 4.86	1.23	−6.67 ^††^ (−10.24, −3.09)		
**AOFAS Score-alignment**						
Intervention Group	0.00 ± 0.00	4.00 ± 4.13	0.97	−4.00 ^†^ (−6.20, −1.80)	−4.00 ^‡‡^ (−6.18, −1.82)	0.001 **
Control Group	0.00 ± 0.00	0.00 ± 0.00	0.00	0.00 (0.00, 0.00)		

^a^ Values are expressed as mean ± standard deviation; ^b^ Values are expressed as mean (95% confidence interval); AOFAS, American orthopedic foot and ankle society; CI, 95% confidence interval; T*G, time and group interaction; Significant differences (^**^ *p* < 0.01, *** *p* < 0.001) in time and group intervention; Significant differences (^†^ *p* < 0.05, ^††^ *p* < 0.01, ^†††^ *p* < 0.001) in within group; Significant differences (^‡‡^ *p* < 0.01) in between groups.

**Table 3:** The changes in pain intensity and PPT (pain pressure threshold).

	Pre ^a^	Post ^a^	Within Group ^b^		Between Groups ^b^	*p*-Value (T*G)
			Effect Size	Mean (CI)		
**VAS-Resting Pain**						
Intervention Group	11.44 ± 10.39	1.25 ± 3.42	−1.11	10.19 ^††^ (4.59, 15.78)	10.99 ^‡‡^ (3.08, 18.89)	0.008 ^**^
Control Group	7.00 ± 11.08	7.80 ± 10.75	0.073	−0.8 (−6.91, 5.31)		
**VAS-Movement Pain**						
Intervention Group	40.63 ± 10.13	7.94 ± 12.21	−2.89	32.69 ^†††^ (25.91, 39.46)	26.56 ^‡‡‡^ (16.84, 36.40)	<0.001 ^***^
Control Group	37.53 ± 19.06	31.46 ± 19.07	−0.32	6.13 (−1.64, 13.77)		
**PPT**						
Intervention Group	5.20 ± 2.50	6.14 ± 2.05	0.41	−0.94 (−2.02, 0.14)	−0.13 (−1.84, 1.59)	0.883
Control Group	5.90 ± 1.89	6.71 ± 2.26	0.39	−0.81 (−2.27, 0.64)		

^a^ Values are expressed as mean ± standard deviation; ^b^ Values are expressed as mean (95% confidence interval); CI, 95% confidence interval; PPT, pressure pain threshold; T*G, time and group interaction; VAS, visual analogue scale; Significant differences (** *p* < 0.01, *** *p* < 0.001) in time and group intervention; Significant differences (^††^ *p* < 0.01, ^†††^ *p* < 0.001) in within group; Significant differences (^‡‡^ *p* < 0.01, ^‡‡‡^ *p* < 0.001) in between groups.

**Table 4:** The changes in ankle ROM (range of motion).

	Pre ^a^	Post ^a^	Within Group ^b^		BetweenGroups ^b^	*p*-Value(T*G)
			Effect Size	Mean (CI)		
**Dorsiflexion**						
Intervention Group	34.26 ± 8.28	41.02 ± 4.97	0.94	−6.76 ^††^ (−11.30, −2.21)	−7.15 ^‡‡‡^ (−12.05, −2.23)	0.006 **
Control Group	32.59 ± 4.04	32.20 ± 5.31	−0.08	0.39 (−1.73, 2.51)		
**Plantar Flexion**						
Intervention Group	30.89 ± 11.36	29.85 ± 7.55	−0.10	1.04 (−3.09, 5.18)	−0.52 (−7.36, 6.33)	0.879
Control Group	28.82 ± 8.51	27.26 ± 6.29	−0.20	1.56 (−4.39, 7.50)		
**Inversion**						
Intervention Group	43.98 ± 8.82	47.67 ± 6.49	0.47	−3.69 (−7.64, 0.26)	−4.63 (−10.07, 0.81)	0.092
Control Group	40.01 ± 8.67	39.07 ± 7.19	−0.12	0.94 (−3.15, 5.02)		
**Eversion**						
Intervention Group	23.85 ± 6.38	28.12 ± 8.38	0.56	−4.27 ^††^ (−6.96, −1.58)	−4.27 ^‡‡^ (−7.79, −0.53)	0.026 *
Control Group	22.15 ± 6.29	22.26 ± 5.60	0.02	0.00 (−2.78, 2.56)		

^a^ Values are expressed as mean ± standard deviation; ^b^ Values are expressed as mean (95% confidence interval); CI, 95% confidence interval; ROM, range of motion; T*G, time and group interaction; Significant differences (* *p* < 0.05, ** *p* < 0.01) in time and group intervention; Significant differences (^††^ *p* < 0.01) in within group; Significant differences (^‡‡^ *p* < 0.01, ^‡‡‡^ *p* < 0.001) in between groups.

**Table 5:** The changes in balance ability.

	Pre ^a^	Post ^a^	Within Group ^b^		Between Groups ^b^	*p*-Value (T*G)
			Effect Size	Mean (CI)		
**Bipedal Stance**						
**Area (cm^2^)**						
Intervention Group	1.83 ± 1.88	1.67 ± 1.44	−0.09	0.16 (−1.15, 1.47)	0.61 (−0.79, 2.01)	0.381
Control Group	1.25 ± 0.87	1.70 ± 1.22	0.41	−0.45 (−1.02, 0.12)		
**Path Length (cm)**						
Intervention Group	12.41 ± 3.39	11.77 ± 1.77	−0.22	0.70 (−0.90, 2.19)	1.80 (−0.22, 3.66)	0.080
Control Group	10.69 ± 2.75	11.77 ± 2.60	0.40	−1.10 (−2.36, 0.21)		
**Velocity (cm/s)**						
Intervention Group	1.24 ± 0.34	1.18 ± 0.18	−0.20	0.06 (−0.09, 0.22)	0.17 (−0.02, 0.37)	0.079
Control Group	1.07 ± 0.28	1.18 ± 0.26	0.40	−0.11 (−0.24, 0.02)		
**APSD**						
Intervention Group	0.16 ± 0.09	0.18 ± 0.10	0.21	−0.02 (−0.10, 0.05)	0.03 (−0.06, 0.12)	0.542
Control Group	0.15 ± 0.09	0.20 ± 0.08	0.59	−0.05 (−0.10, 0.01)		
**LRSD**						
Intervention Group	0.17 ± 0.11	0.14 ± 0.07	−0.31	0.03 (−0.02, 0.09)	0.03 (−0.05, 0.13)	0.338
Control Group	0.11 ± 0.05	0.11 ± 0.12	0.00	0.00 (−0.08, 0.06)		
**Unipedal Stance**						
**Area (cm^2^)**						
Intervention Group	8.93 ± 3.74	7.67 ± 2.69	−0.38	1.26 (−0.65, 3.15)	0.12 (−2.56, 2.80)	0.928
Control Group	7.46 ± 4.20	6.32 ± 2.72	−0.30	1.14 (−0.93, 3.21)		
**Path Length (cm)**						
Intervention Group	51.79 ± 11.83	40.49 ± 11.51	−0.97	11.30 ^††^ (4.73, 17.88)	9.90 ^‡‡^ (3.11, 16.83)	0.006 **
Control Group	47.90 ± 12.49	46.56 ± 12.56	−0.11	1.40 (−0.96, 3.63)		
**Velocity (cm/s)**						
Intervention Group	5.18 ± 1.18	4.05 ± 1.15	−0.97	1.13 ^††^ (0.47, 1.79)	1.00 ^‡‡^ (0.31, 1.68)	0.006 **
Control Group	4.79 ± 1.25	4.66 ± 1.26	−0.11	0.13 (−0.10, 0.37)		
**APSD**						
Intervention Group	0.46 ± 0.17	0.32 ± 0.10	−0.95	0.14 ^†^ (0.03, 0.24)	0.10 (−0.04, 0.24)	0.140
Control Group	0.41 ± 0.19	0.37 ± 0.11	−0.24	0.04 (−0.06, 0.14)		
**LRSD**						
Intervention Group	0.45 ± 0.19	0.36 ± 0.11	−0.55	0.09 (−0.01, 0.20)	0.08 (−0.11, 0.27)	0.392
Control Group	0.43 ± 0.19	0.42 ± 0.20	−0.05	0.01 (−0.16, 0.19)		

^a^ Values are expressed as mean ± standard deviation; ^b^ Values are expressed as mean (95% confidence interval); APSD, anteroposterior standard deviation; CI, 95% confidence interval; LRSD, left-right standard deviation; T*G, time and group interaction; Significant differences (** *p* < 0.01) in time and group intervention; Significant differences (^†^ *p* < 0.01; ^††^ *p* < 0.01) in within group; Significant differences (^‡‡^ *p* < 0.01) in between groups.
